# Supplementary material for: Effects of multi-functional additives during foam extrusion of wheat gluten materials
Source: Commun Chem. 2024 Apr 3;7:75. doi: 10.1038/s42004-024-01150-1 (PMC10991538; doi:10.1038/s42004-024-01150-1)
Supplement: Supplementary file 2 — Supplementary Information [file 42004_2024_1150_MOESM2_ESM.pdf]

Supplementary information

Effects of multi-functional additives during foam extrusion of wheat gluten materials

Mercedes A. Bettelli,[1] Qisong Hu [1], Antonio J. Capezza [1], Eva Johansson, [2] Richard T. Olsson,[1] Mikael S. Hedenqvist,[1]*

1 Department of Fibre and Polymer Technology, Polymeric Materials Division, School of Engineering Sciences in Chemistry, Biotechnology and Health. KTH Royal Institute of Technology, Stockholm 10044, Sweden

2 Department of Plant Breeding, The Swedish University of Agricultural Sciences, Box 190, SE-234 22 Lomma, Sweden

* Correspondence: mikaelhe@kth.se; Tel: +46-706507645.

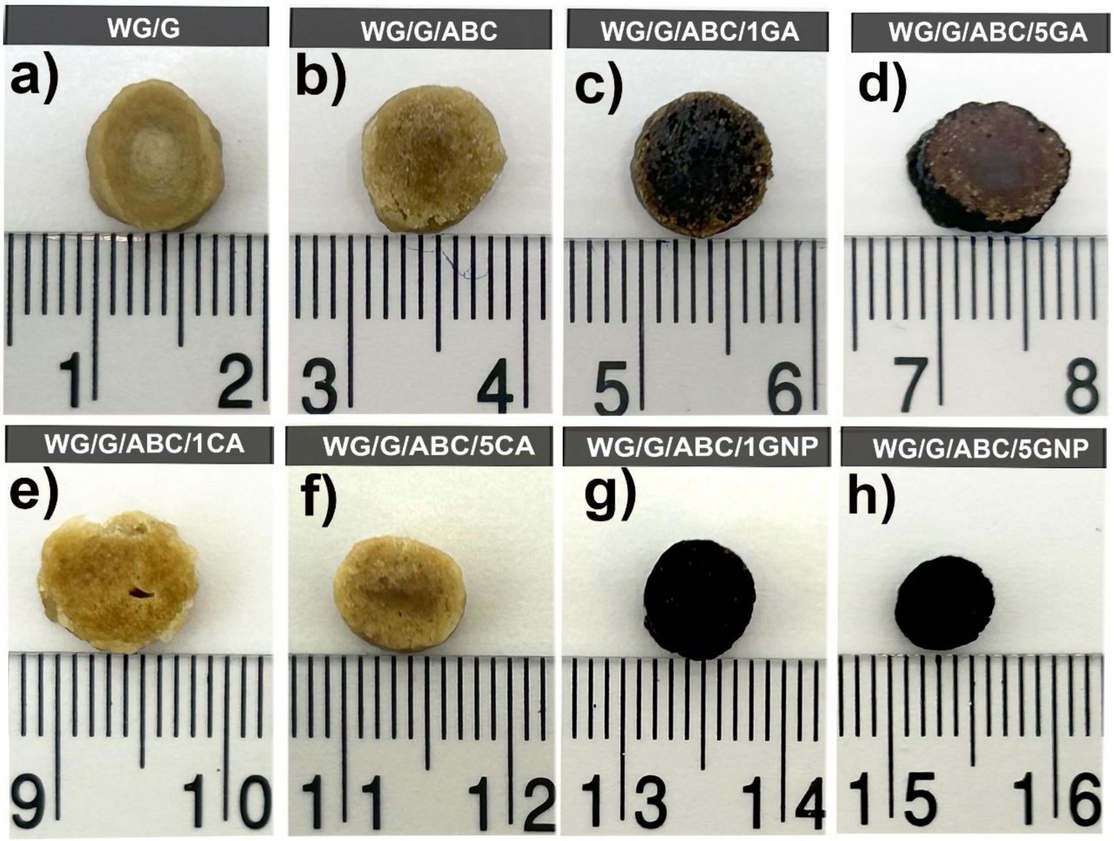

Supplementary Fig. S1. Colour gradient of the cross-section of wheat gluten the foams**. a,** WG/G, **b,** WG/G/ABC, **c,** WG/G/ABC/1GA, **d**, WG/G/ABC/5GA, **e**, WG/G/ABC/1CA, **f**, WG/G/ABC/5GA, **g**, WG/G/ABC/1GNP and **h**, WG/G/ABC/5GNP.

Supplementary Table S1. Theoretical calculation of the foaming power of ammonium bicarbonate.

Sample	Blowing agent (wt.%)	Exposure time (min)	Theoretical gas volume (mL)	Volume of gas displacement (mL)	Total gas volume (mL/2.5 g ABC)	Decomposition rate (DR) (mL/min)
**ABC**	100	5	2530	100	40	20.0
		8		200	80	
**WG/G**	0	5		-	-	-
		10		5	-	0.5
**WG/G/ABC**	5	10		30	10	3.0
		20		80	30	4.0

Note: The amount of ABC was 2.5 grams, which is equivalent to 0.032 moles, yielding a total gas generation of 40 mL in 5 min, considering the ammonia released. The gas volume was calculated based on the stoichiometry, including the volume of each gas generated during the decomposition process (NH_3, CO_2, and H_2O). The temperature used was 70 °C at 1 atmosphere pressure.

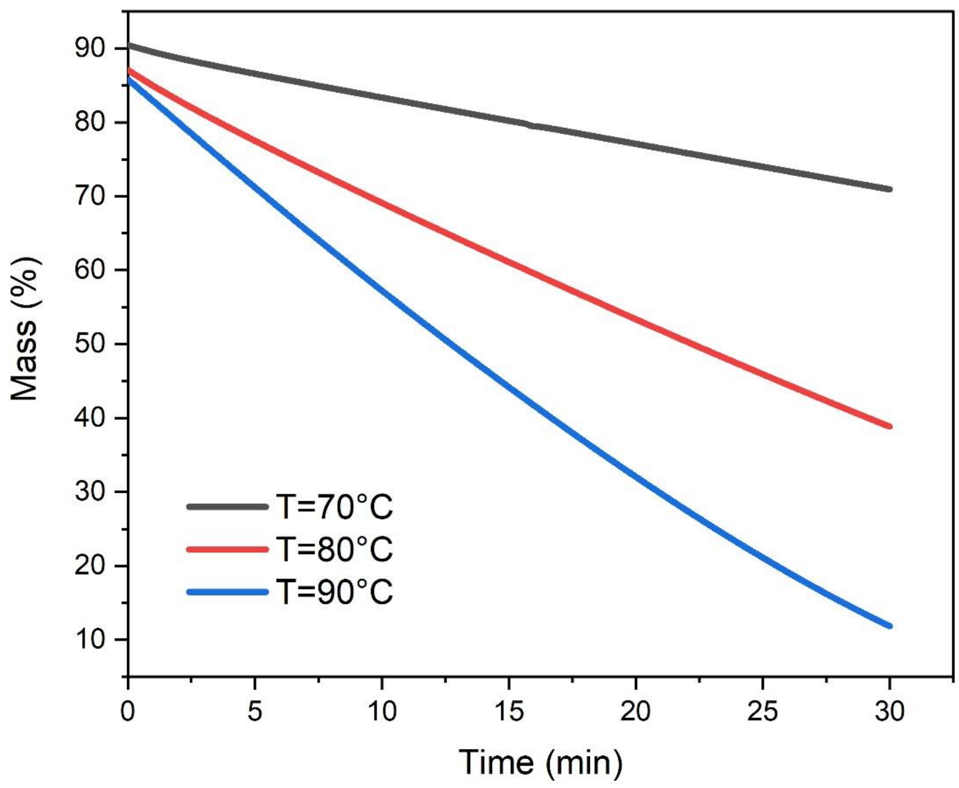

Supplementary Fig. S2. Ammonium bicarbonate at different decomposition temperatures.

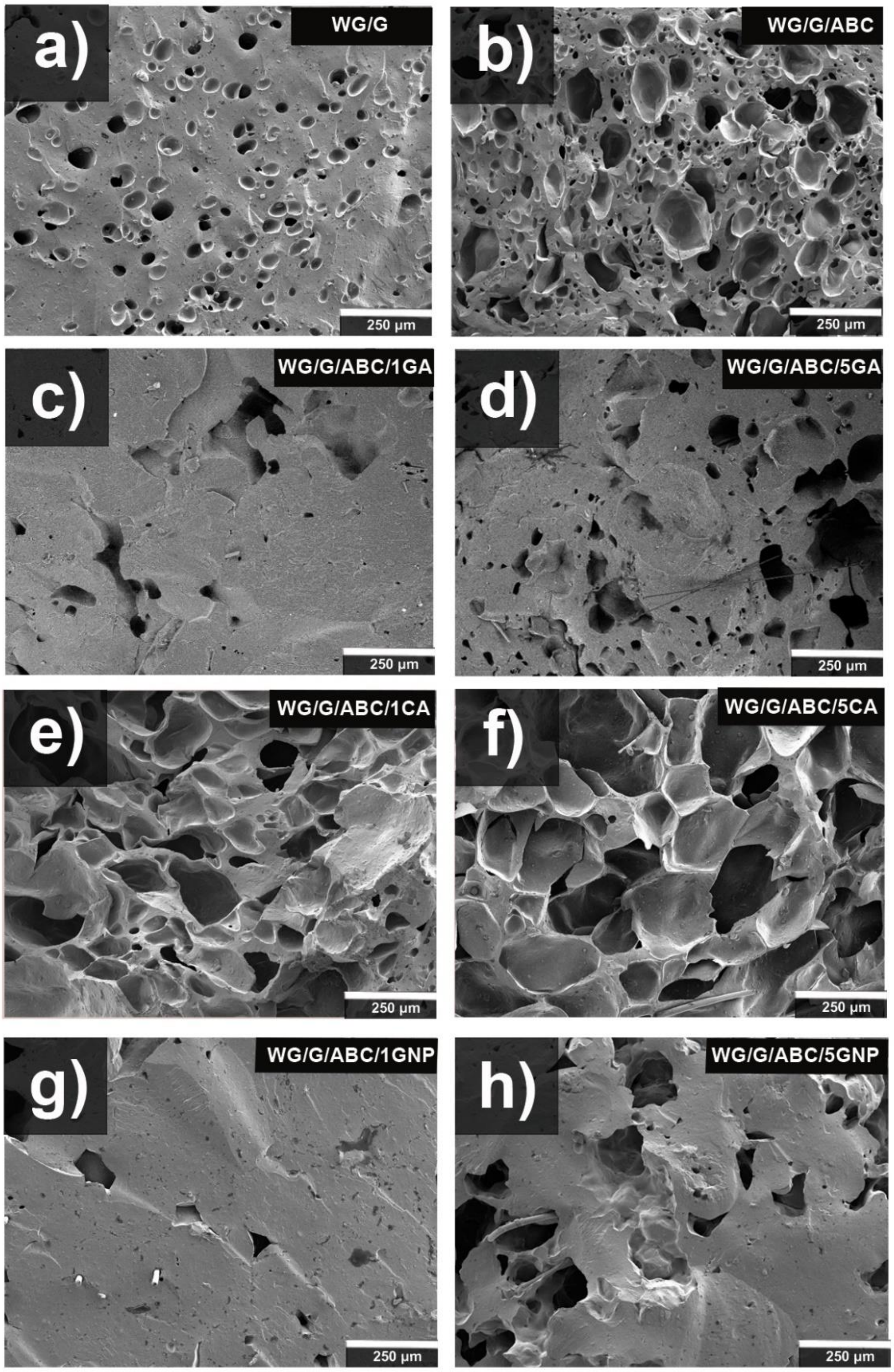

Supplementary Fig. S3. High-magnification FE-SEM micrographs of the foams.

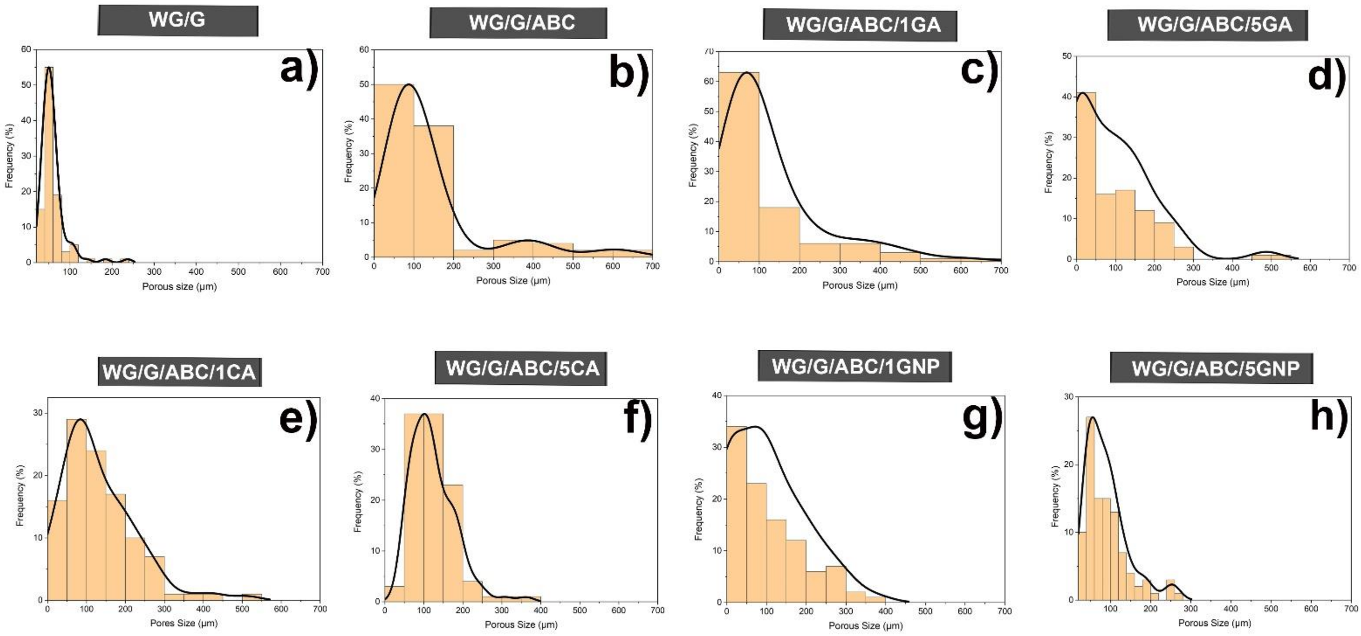

Supplementary Fig. S4. Pore size distribution of the foams.

a)

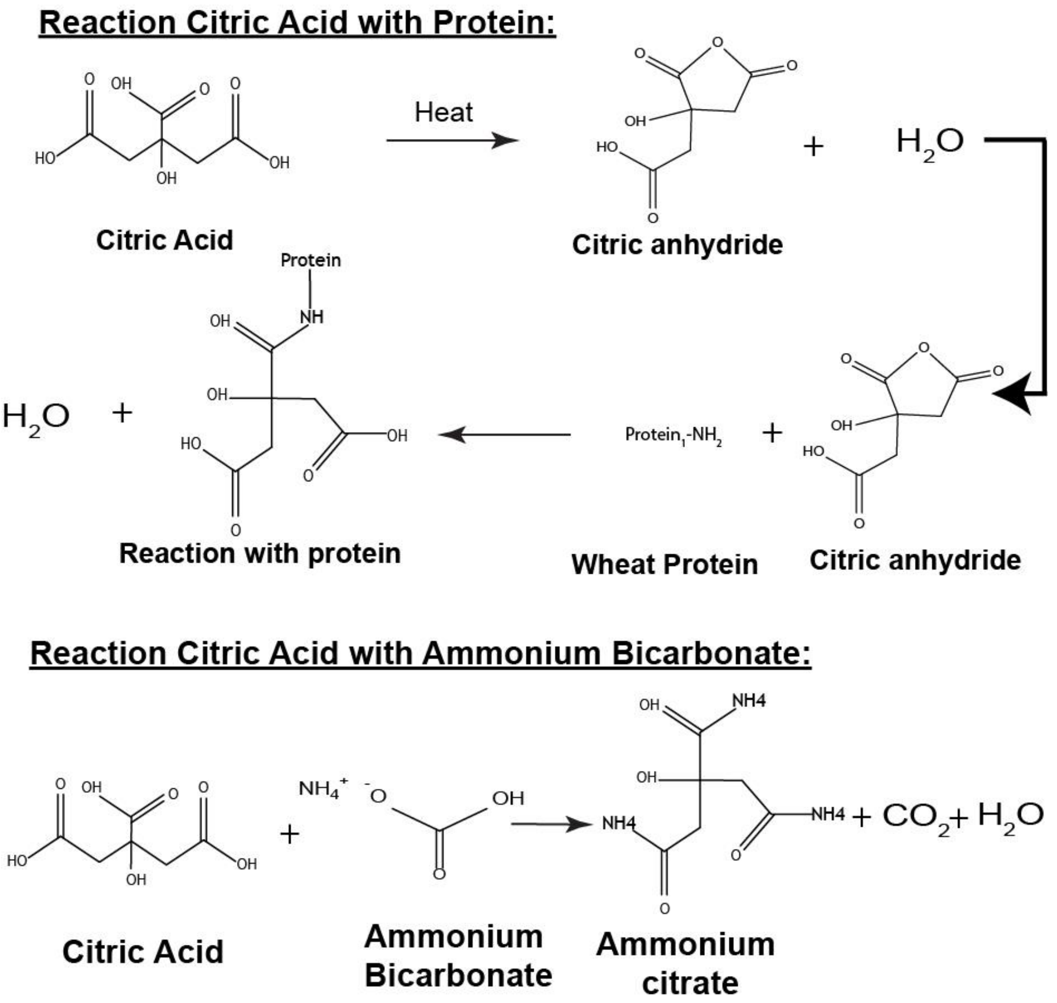

Reaction Citric Acid with Protein:
Heat
Citric Acid
Citric anhydride
+
H_2O
Protein
NH
OH
HO
O
H_2O +
Protein$_1$-NH_2
+
Reaction with protein
Wheat Protein
Citric anhydride
Reaction Citric Acid with Ammonium Bicarbonate:
NH_4^+
NH4
+ CO_2 + H_2O
+
Citric Acid
Ammonium Bicarbonate
Ammonium citrate

b)

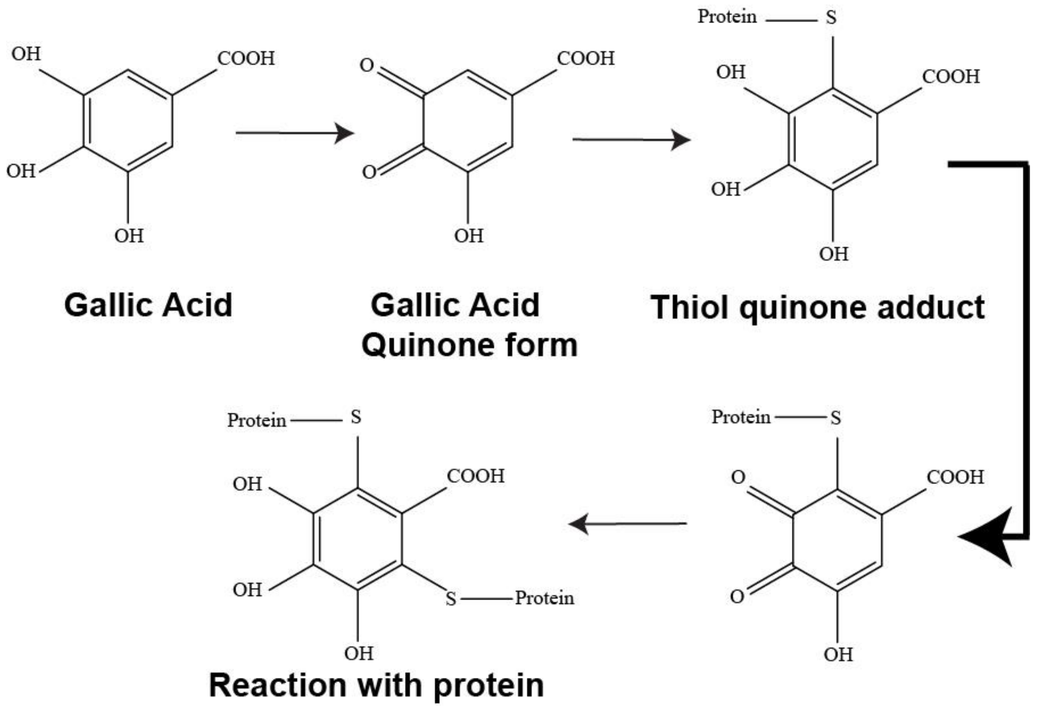

OH
COOH
O
Protein — S
Gallic Acid
Gallic Acid Quinone form
Thiol quinone adduct
S — Protein
Reaction with protein

c)

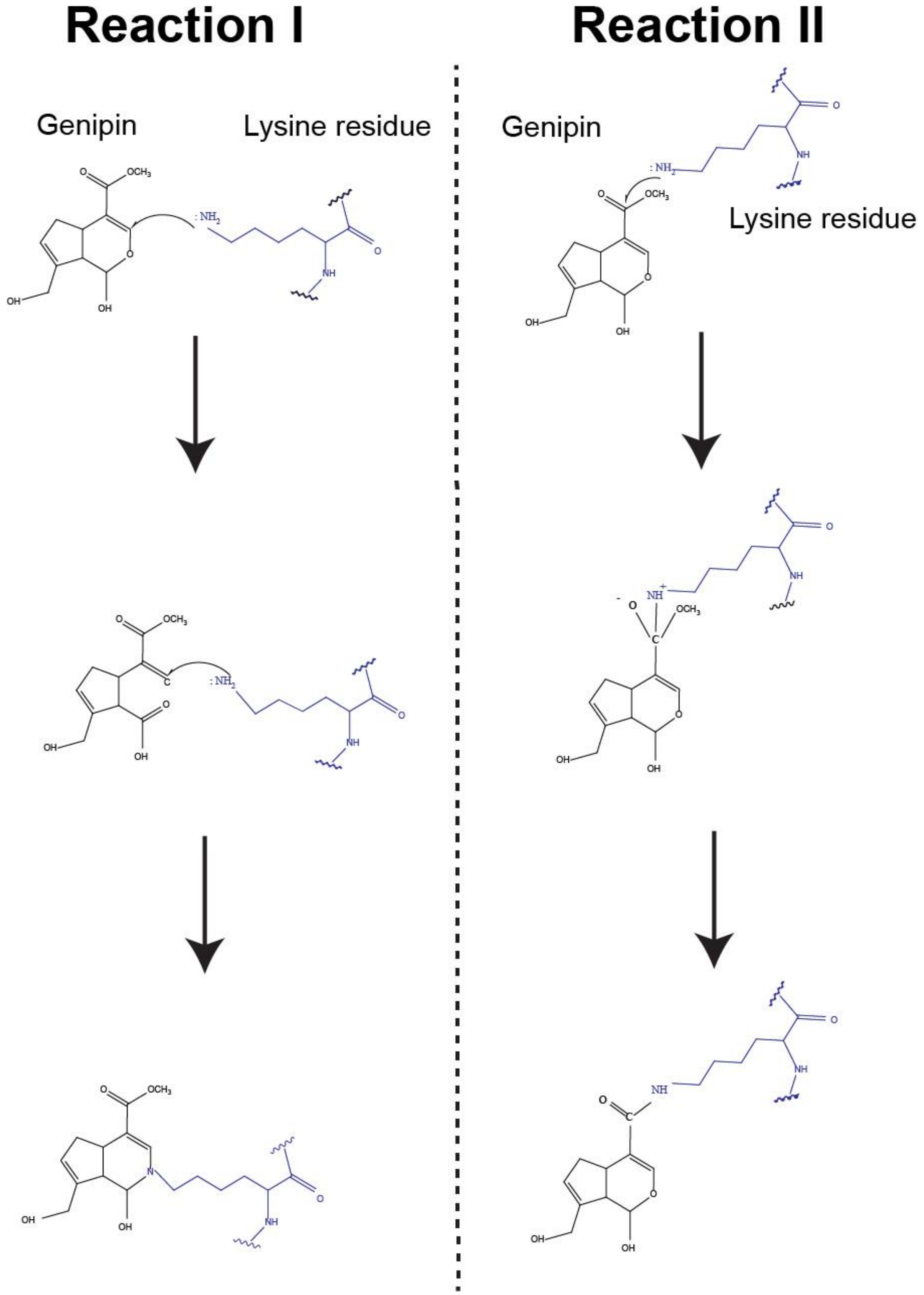
Reaction I
Reaction II
Genipin
Lysine residue
Genipin
Lysine residue

d)

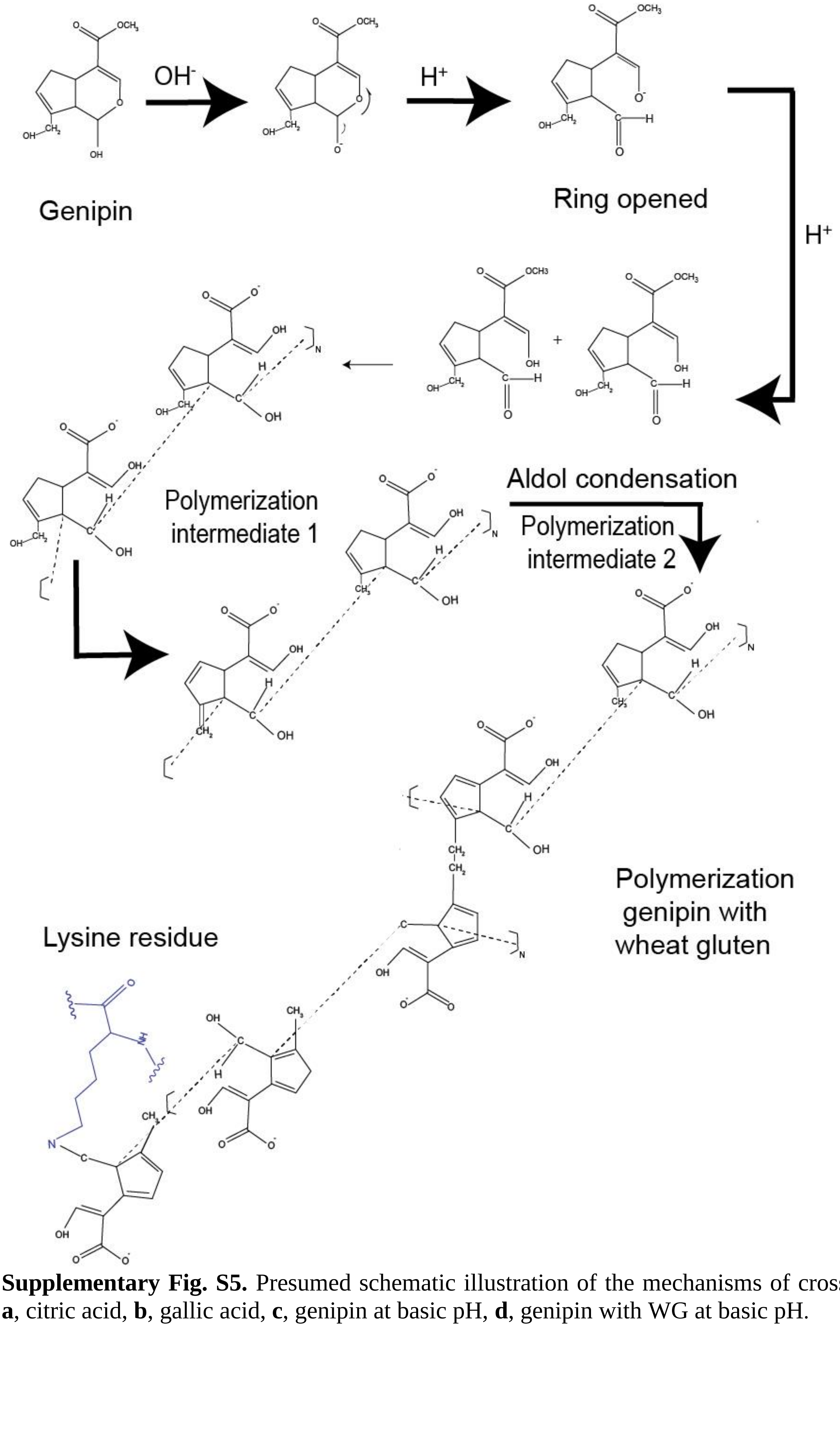

Supplementary Fig. S5. Presumed schematic illustration of the mechanisms of crosslinking. **a**, citric acid, **b**, gallic acid, **c**, genipin at basic pH, **d**, genipin with WG at basic pH.

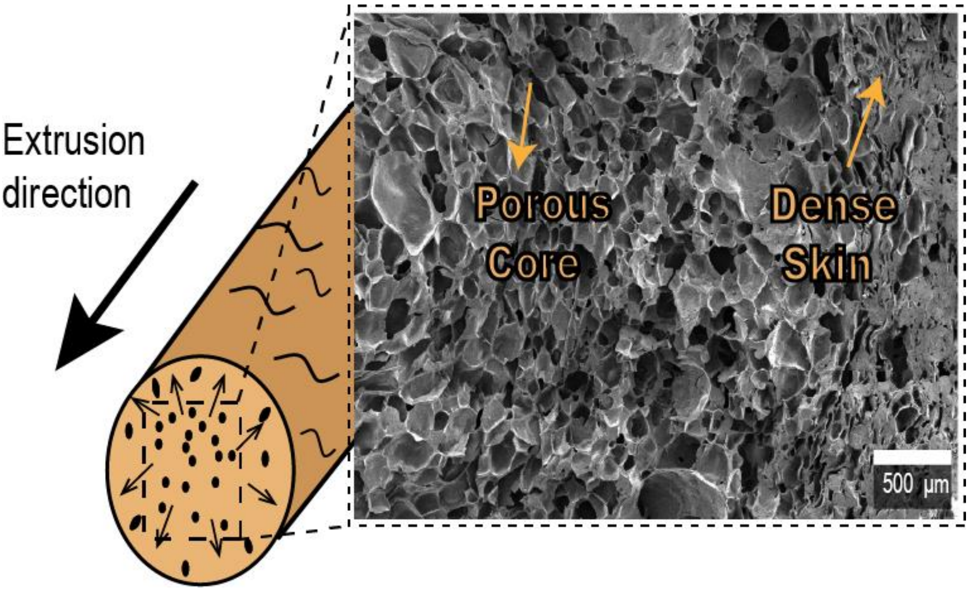

Supplementary Fig. S6. Cross-section of the WG/G/ABC/5CA foam extrudate.

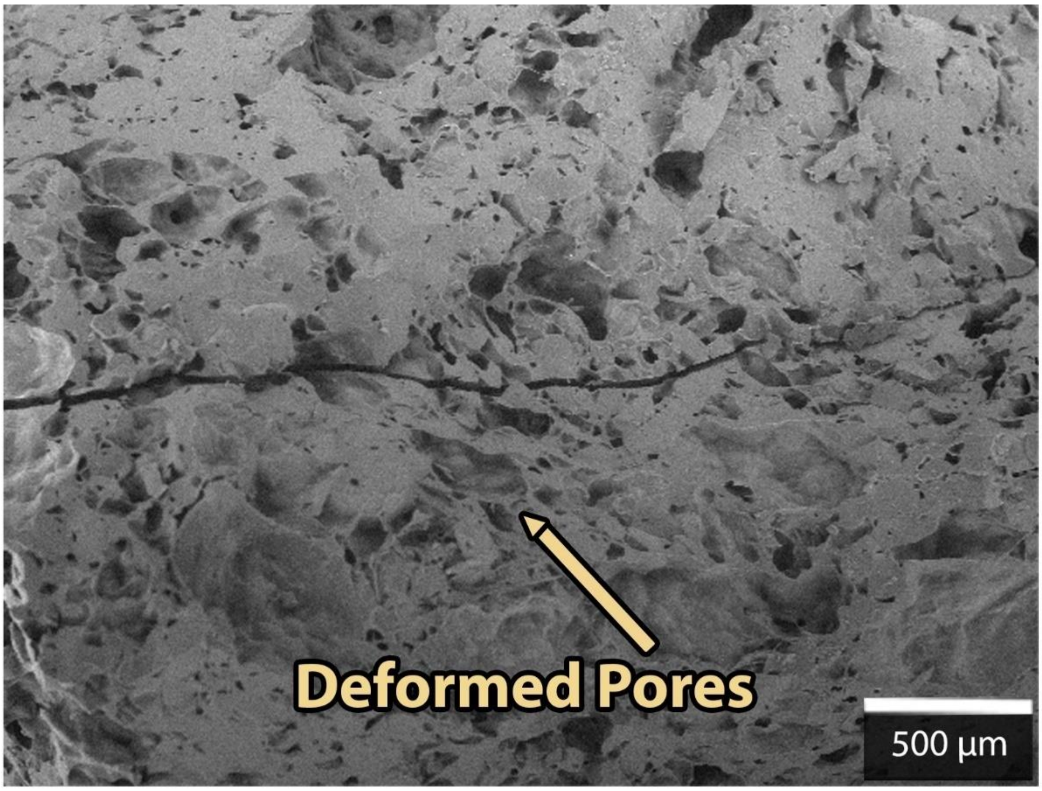

Supplementary Fig. S7. Transverse-section (cut along the extrusion direction) of the WG/G/ABC/5CA foam extrudate.

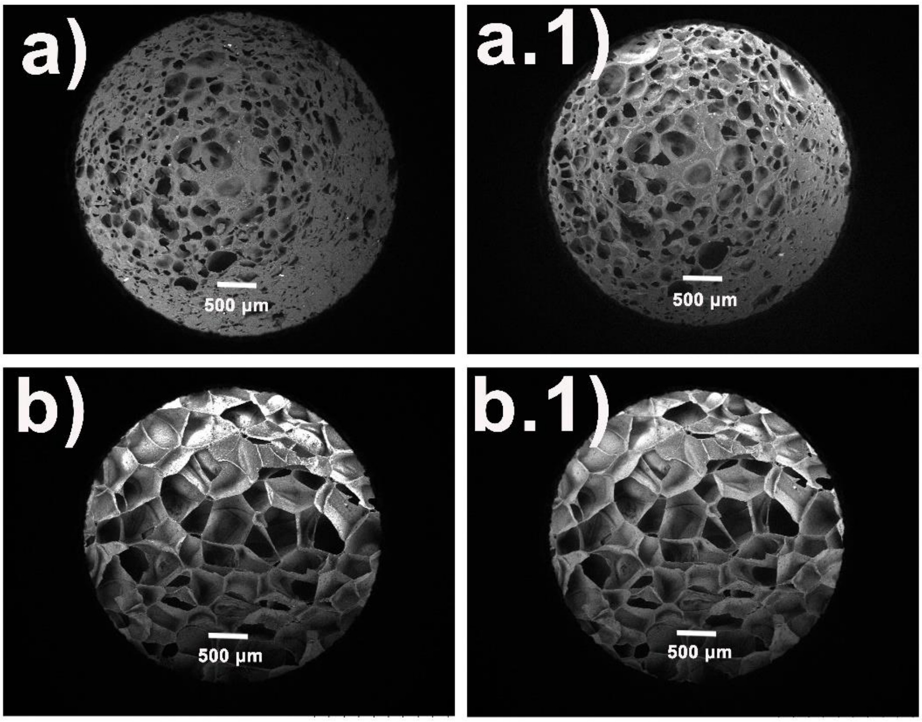

Supplementary Fig. S8. SEM images of WG/G/ABC/5CA and NBR (**b, b.1**) foams at 10 % (**a,b**) and 50 % (**a.1,b.1**) compressive strain.

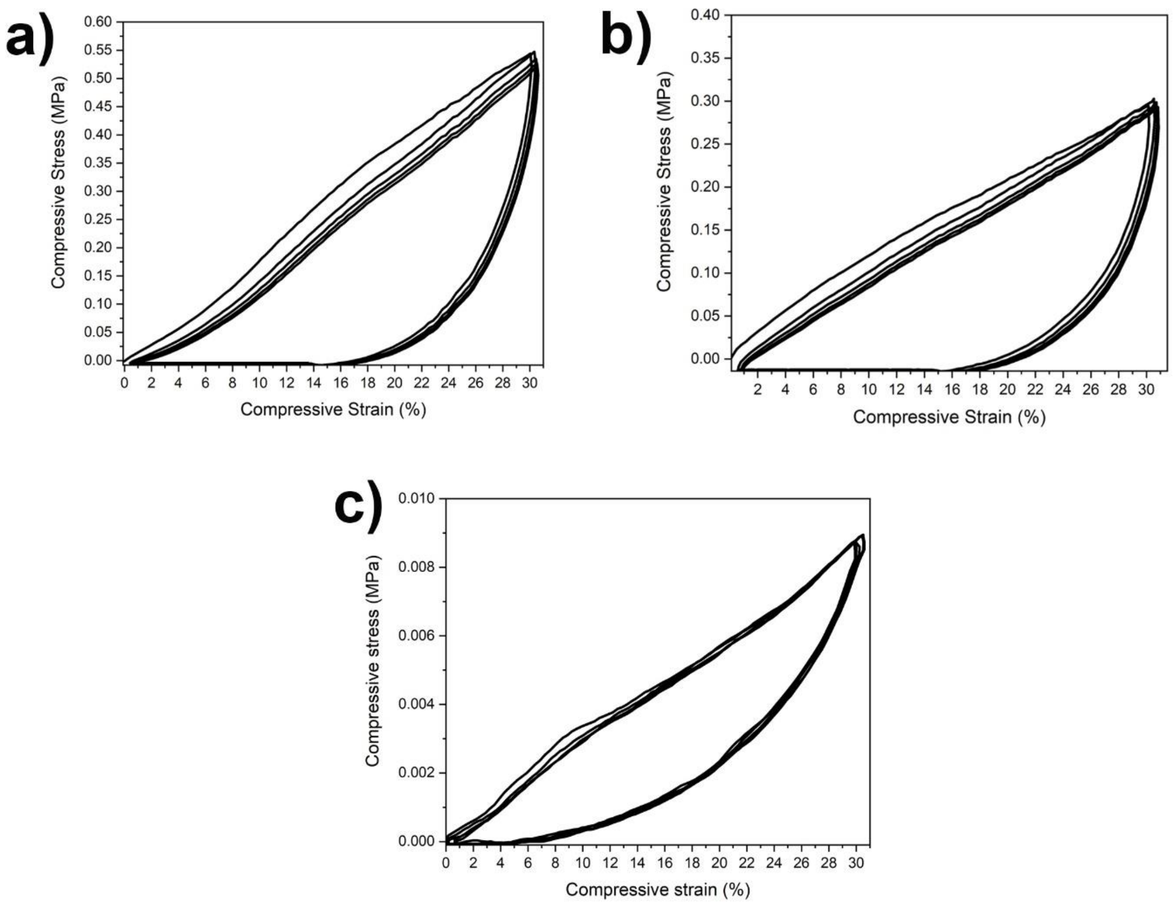

Supplementary Fig. S9. Representative compression curves at the same repetitive compression strain interval (0-30 %). **a**, WG/G, **b**, WG/G/ABC/5CA, and **c**, NBR foams.

Supplementary Table S2. Mechanical properties of the samples at 30% strain.

				**WG/G**					
**$\sigma_{y10\%}$[1] (kPa)**	**$\sigma_{30\%}$[2] $_{1Cycle}$(kPa)**	**$\sigma_{30\%}$ $_{3Cycles}$(kPa)**	**$\sigma_{30\%}$ $_{5Cycless}$(kPa)**	**$E_{30\%}$[3] $_{1Cycle}$(MPa)**	**$E_{30\%}$ $_{3Cycles}$(MPa)**	**$E_{30\%}$ $_{5Cycles}$(MPa)**	**$A_{f30\%}$[4] $_{1Cycle}$(%)**	**$A_{f30\%}$ $_{3Cycles}$(%)**	**$A_{f30\%}$ $_{5Cycles}$(%)**
50±20[c]	600±54[c]	578±50[c]	516±48[c]	2.0±0.6[c]	1.32±0.52[b]	1.19±0.56[b]	81.7±0.8[b]	80.0±0.8[b]	79.0±0.6[b]
				**WG/G/ABC/5CA**					
**$\sigma_{y10\%}$ (kPa)**	**$\sigma_{30\%}$ $_{1Cycle}$(kPa)**	**$\sigma_{30\%}$ $_{3Cycles}$(kPa)**	**$\sigma_{30\%}$ $_{5Cycles}$(kPa)**	**$E_{30\%}$ $_{1Cycle}$(kPa)**	**$E_{30\%}$ $_{3Cycles}$(MPa)**	**$E_{30\%}$ $_{5Cycles}$(MPa)**	**$A_{f30\%}$ $_{1Cycle}$(%)**	**$A_{f30\%}$ $_{3Cycles}$(%)**	**$A_{f30\%}$ $_{5Cycles}$(%)**
19±12[b]	217±53[b]	220±54[b]	230±37[b]	0.74±0.59[b]	0.69±0.55[b]	0.56±0.48[b]	83.0±2.0[b]	82.0±1.4[b]	82.0±1.2[c]
				**NBR**					
**$\sigma_{y10\%}$ (kPa)**	**$\sigma_{30\%}$ $_{1Cycle}$(kPa)**	**$\sigma_{30\%}$ $_{3Cycles}$(kPa)**	**$\sigma_{30\%}$ $_{5Cycles}$(kPa)**	**$E_{30\%}$ $_{1Cycle}$(kPa)**	**$E_{30\%}$ $_{3Cycles}$(MPa)**	**$E_{30\%}$ $_{5Cycles}$(MPa)**	**$A_{f30\%}$ $_{1Cycle}$(%)**	**$A_{f30\%}$ $_{3Cycles}$(%)**	**$A_{f30\%}$ $_{5Cycles}$(%)**
1.1±0.3[a]	9.9±1.5[a]	9.9±1.4[a]	9.8±1.2[a]	0.05±0.03[a]	0.04±0.01[a]	0.04±0.03[a]	56.0±1.7[a]	55.7±2.3[a]	55.5±2.6[a]

Note: Different letters mean the values are significantly different ($P < 0.05$) in each column.

[1] $\sigma_{y10\%}$: Yield strength at 10% of deformation.
[2] $\sigma_{30\%}$: Maximum compression strength at 30% of deformation.
[3] $E_{30\%}$: Elastic moduli at 30% of deformation.
[4] $A_{f30\%}$: Hysteresis loss rate at 30% of deformation.

Supplementary Table S3: Recovery of the samples one month from the compression test.

Time	WG/G	WG/G/ABC/5CA	NBR
1h	99.3±0.9^{a}	97.2±0.5^{a}	99.1±2.0^{a}
5h	97.9±2.6^{A}	95.6±1.0^{A}	96.2±0.8^{A}
24h	92.4±0.8^{II}	89.6±3.4^{II}	84.4±0.3^{I}
48h	84.4±4.3*	82.7±2.6*	80.9±2.0*
1w	72.2±2.3^{i}	75.4±3.2^{i}	81.6±4.2^{ii}

Note: Different superscript letters and symbols mean that the values are significantly different ($P < 0.05$) in each row.

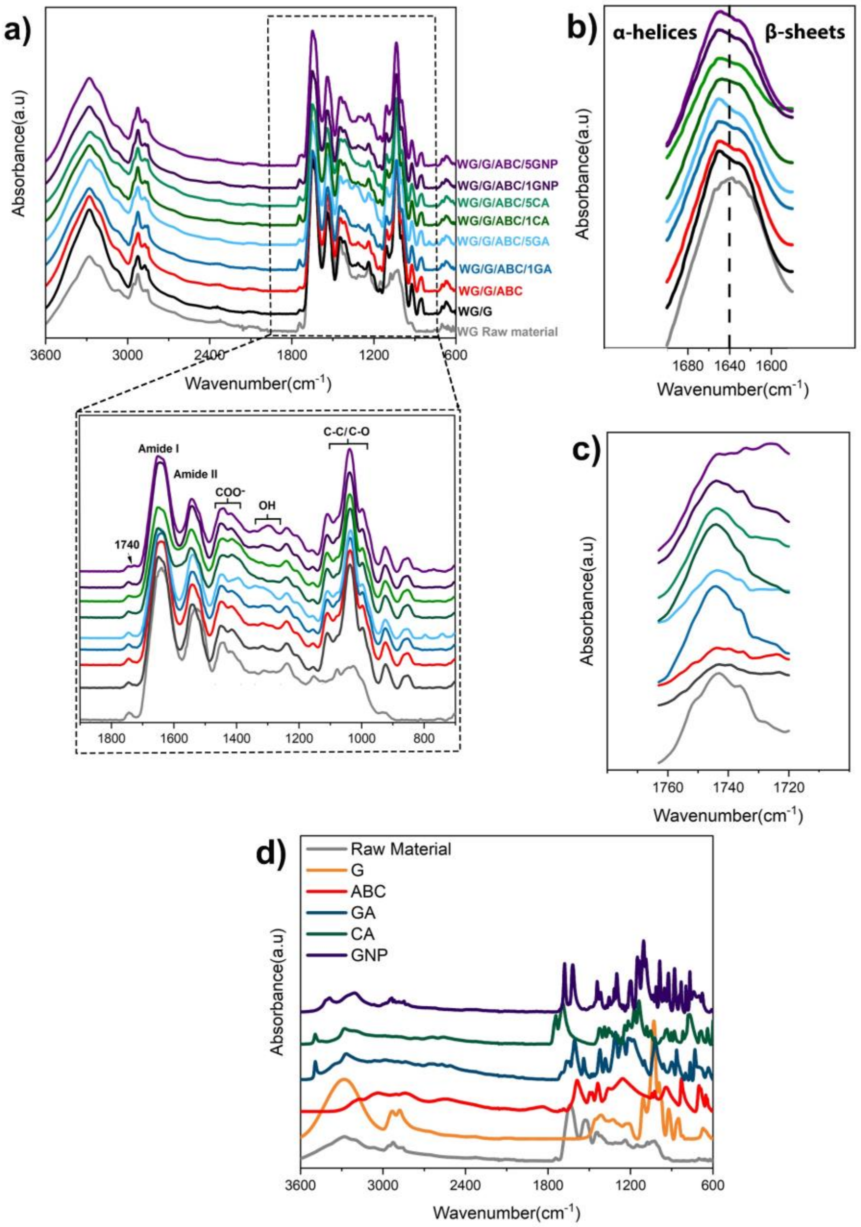

Supplementary Fig. S10. FTIR spectra the WG foams. **a**, Full FTIR spectra and the **b**, amide I region, **c**, 1760-1720 cm^{-1} region and, **d**, Full FTIR spectra for the WG raw material and the additives.

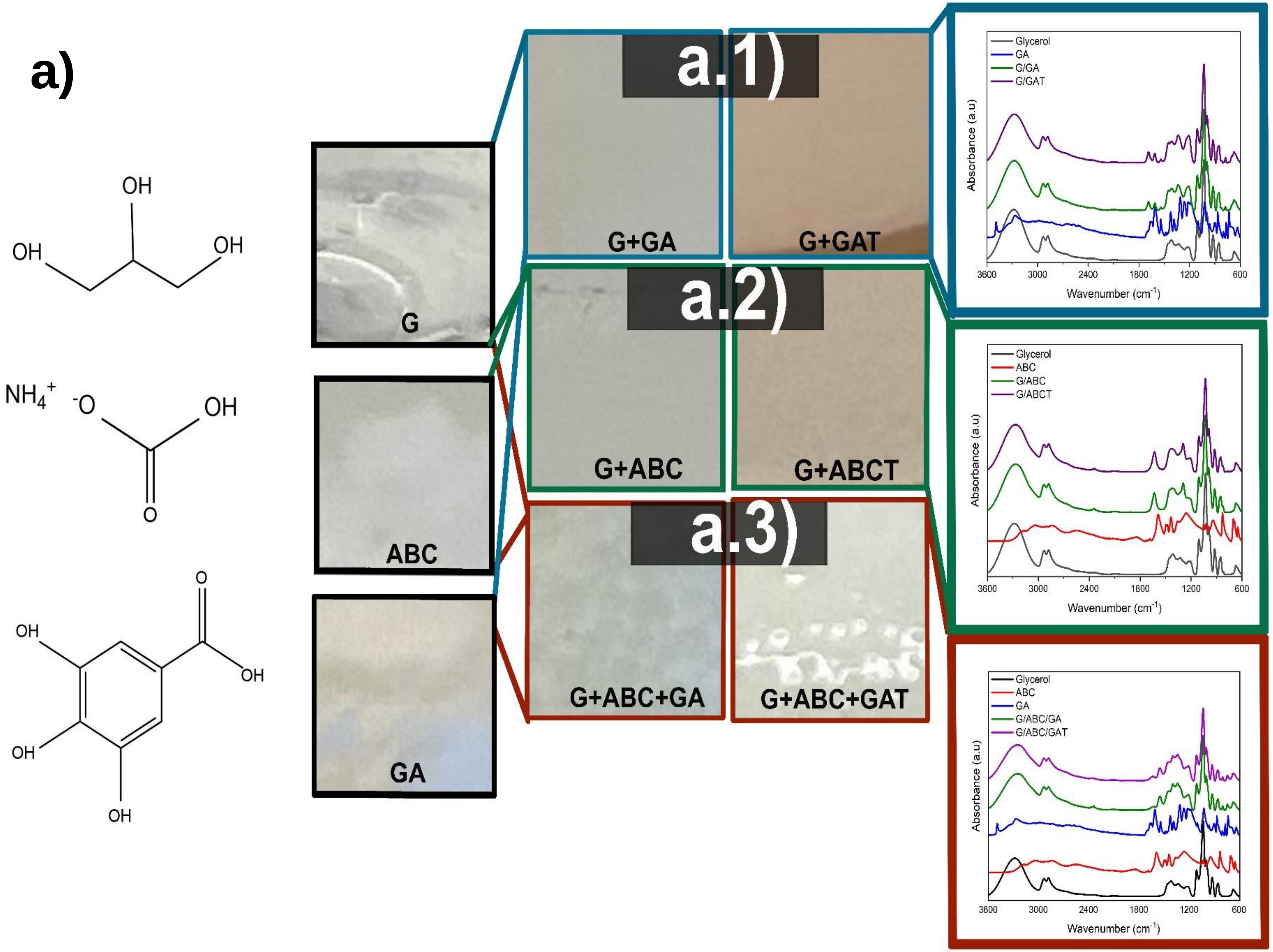
a)
OH
OH
OH
NH4+
-O
OH
O
O
OH
OH
OH
OH
OH
G
ABC
GA
a.1)
G+GA
G+GAT
a.2)
G+ABC
G+ABCT
a.3)
G+ABC+GA
G+ABC+GAT
Glycerol
GA
G/GA
G/GAT
Absorbance (a.u)
Wavenumber (cm-1)
Glycerol
ABC
G/ABC
G/ABCT
Absorbance (a.u)
Wavenumber (cm-1)
Glycerol
ABC
GA
G/ABC/GA
G/ABC/GAT
Absorbance (a.u)
Wavenumber (cm-1)

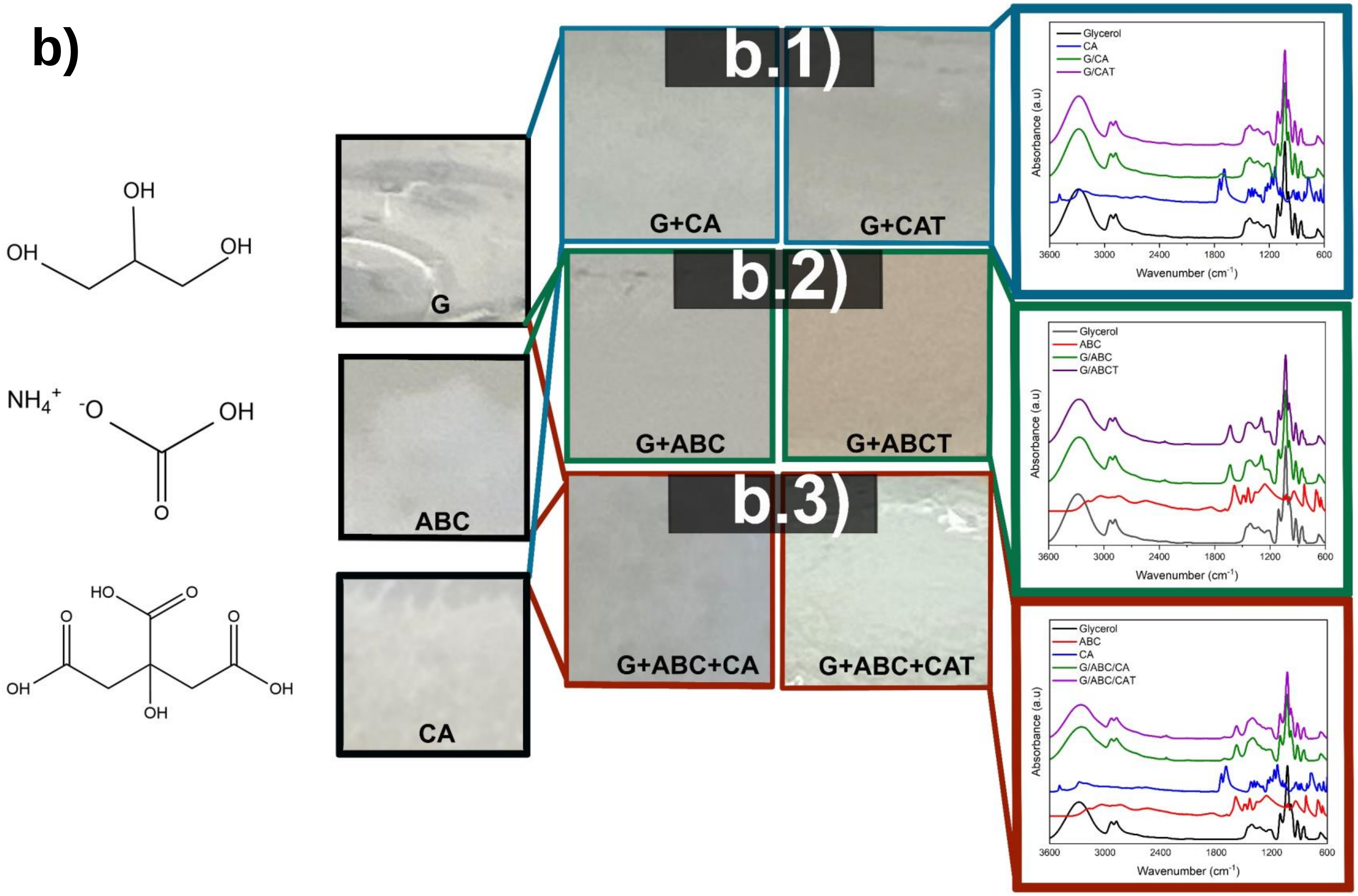
b)
OH
OH
OH
NH_4^+ ^-O OH
O
HO O
O O
OH OH
OH
G
ABC
CA
b.1)
G+CA
G+CAT
b.2)
G+ABC
G+ABCT
b.3)
G+ABC+CA
G+ABC+CAT
Glycerol
CA
G/CA
G/CAT
Absorbance (a.u)
Wavenumber (cm^{-1})
Glycerol
ABC
G/ABC
G/ABCT
Glycerol
ABC
CA
G/ABC/CA
G/ABC/CAT
3600 3000 2400 1800 1200 600

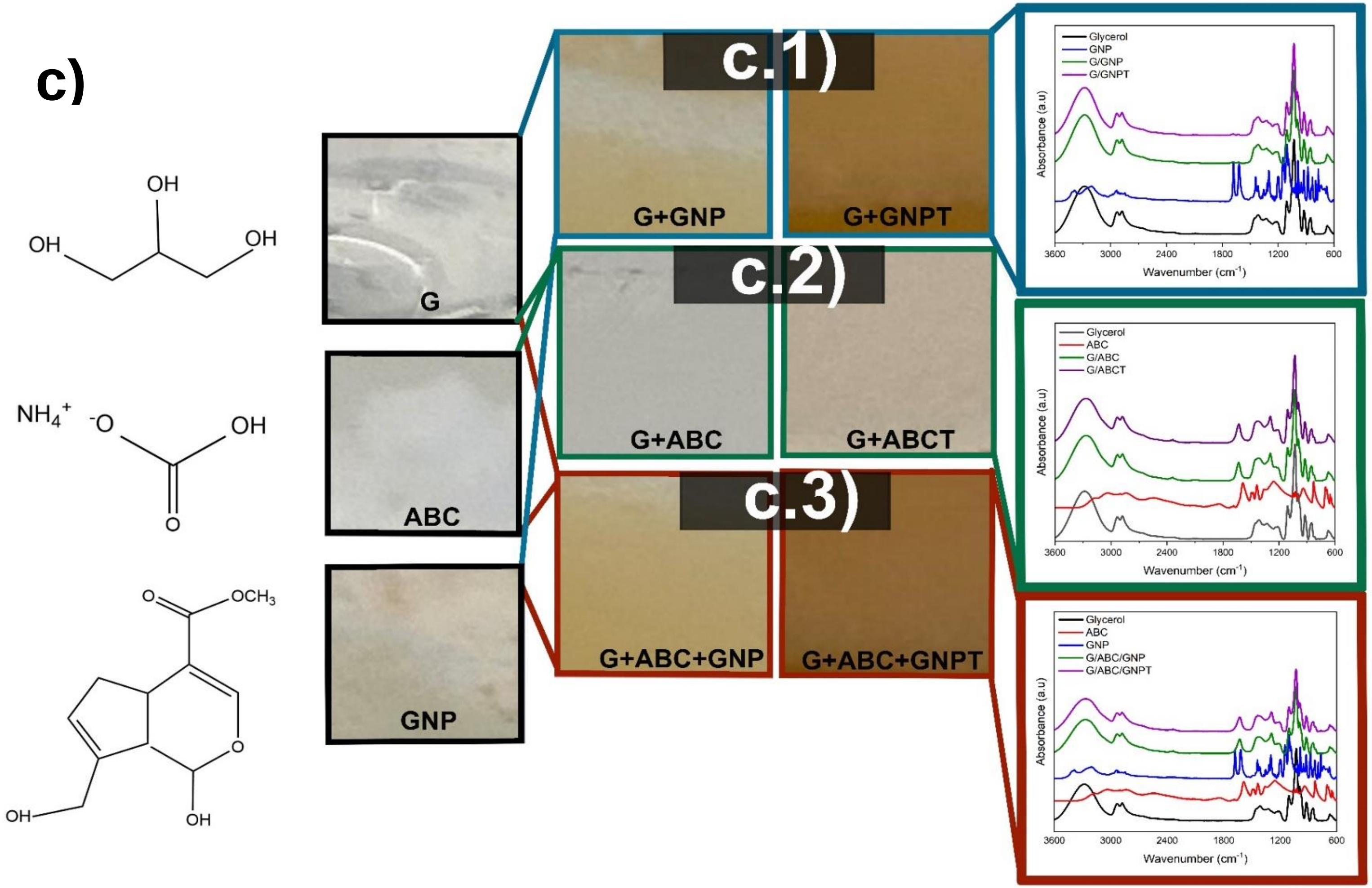

Supplementary Fig. S11. Molecular structure, images illustrating the different reactions in the mixtures and full FTIR spectra. **a**, Gallic acid, **b**, citric acid, and **c**, genipin systems.

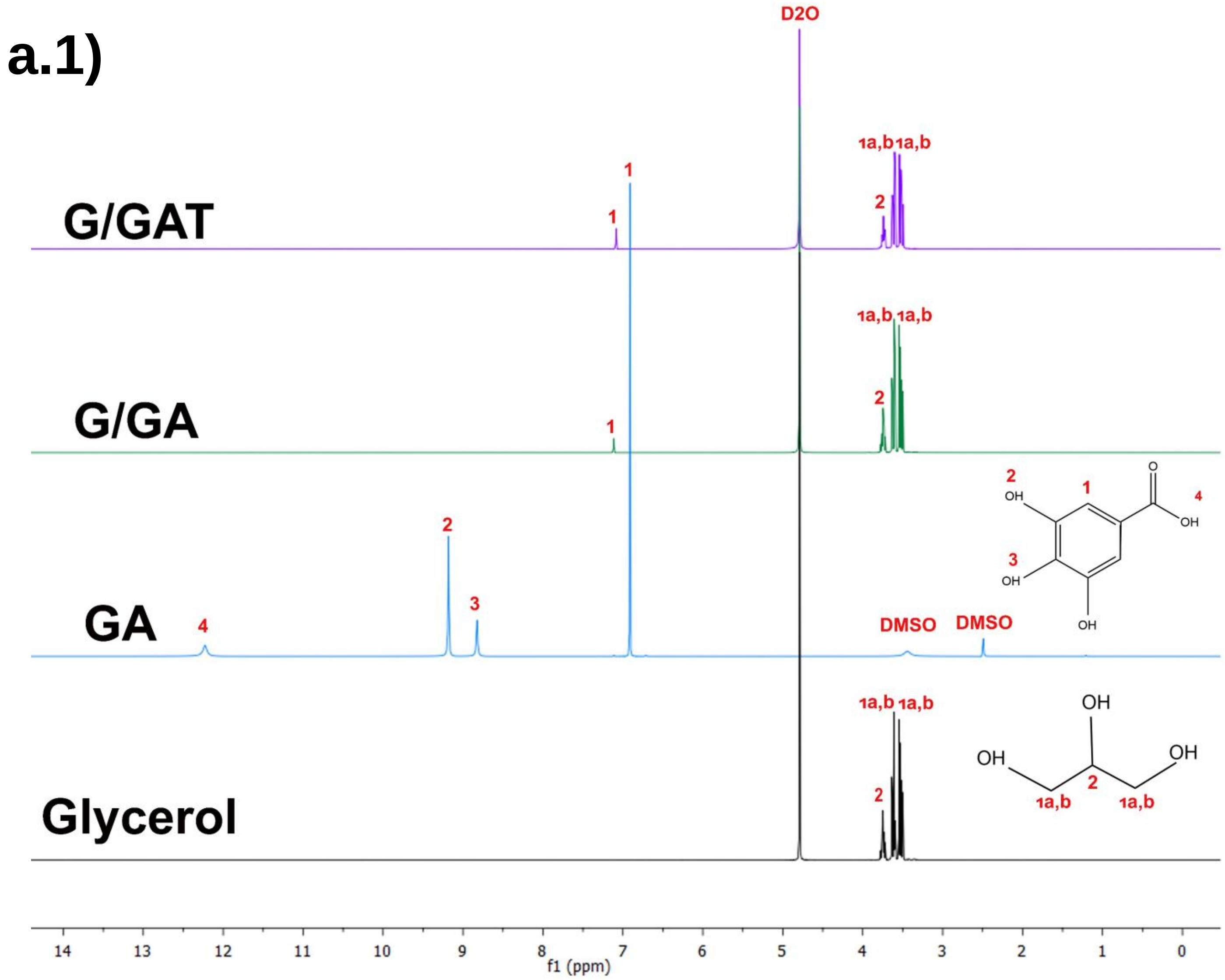
a.1)
D2O
G/GAT
1
1
2
1a,b 1a,b
G/GA
1
2
1a,b 1a,b
GA
4
2
3
DMSO
DMSO
2
OH
1
O
4
OH
3
OH
OH
Glycerol
2
1a,b 1a,b
OH
OH
OH
2
1a,b
1a,b
14 13 12 11 10 9 8 7 6 5 4 3 2 1 0
f1 (ppm)

a.2)

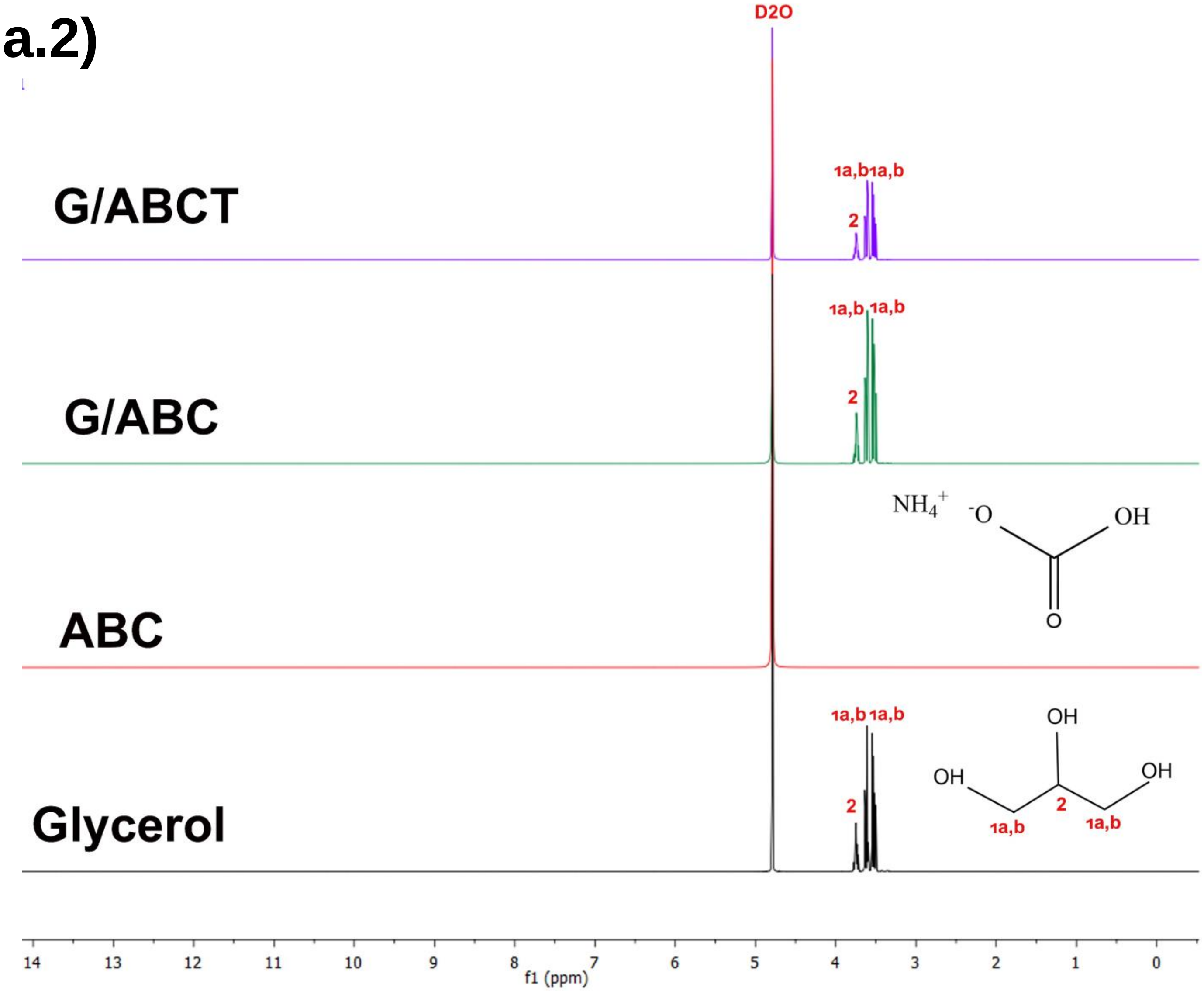
D2O
G/ABCT
1a,b
1a,b
2
G/ABC
1a,b
1a,b
2
NH4+
-O
OH
O
ABC
Glycerol
1a,b
1a,b
2
OH
OH
OH
2
1a,b
1a,b
14
13
12
11
10
9
8
7
6
5
4
3
2
1
0
f1 (ppm)

a.3)

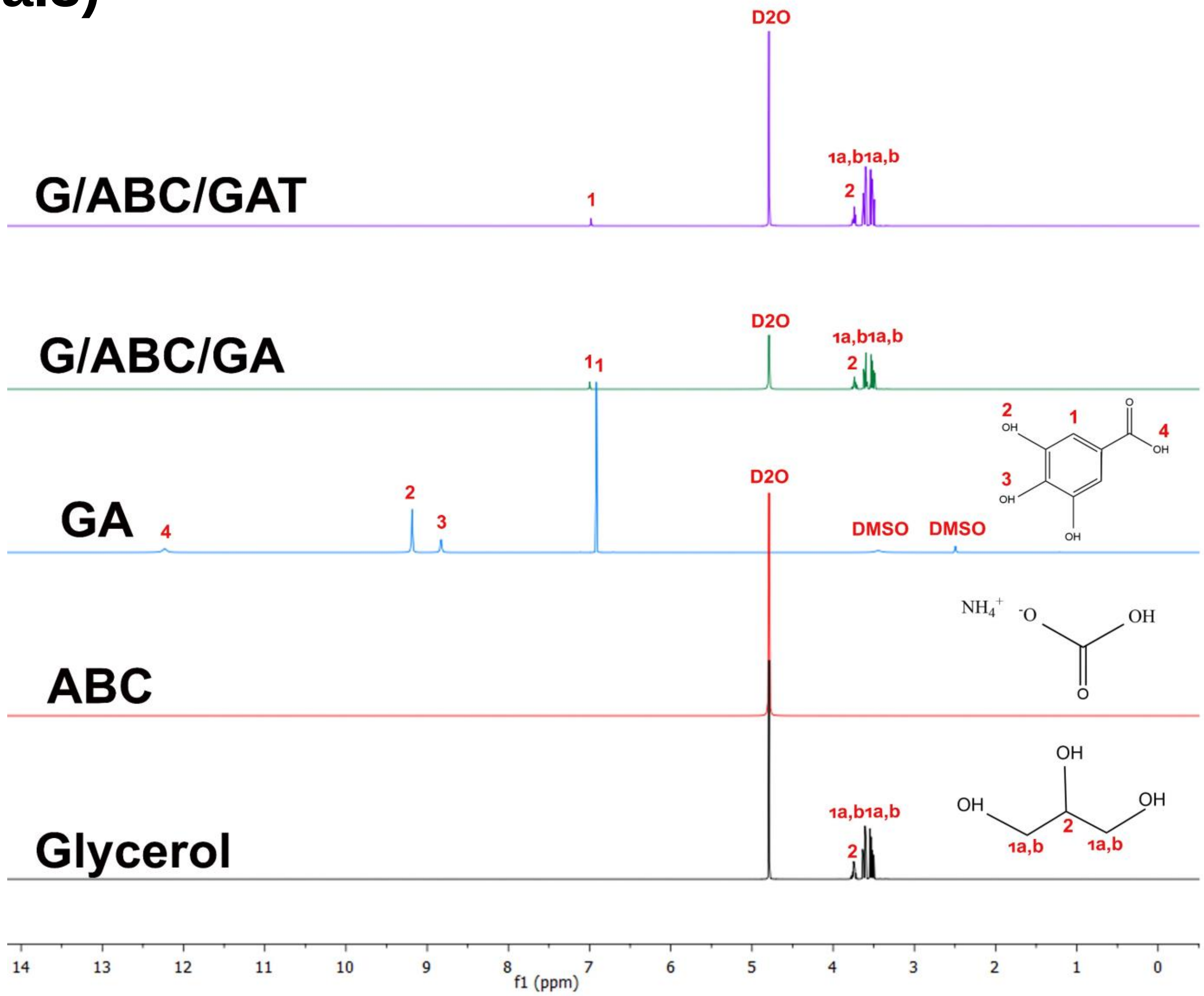
D2O
G/ABC/GAT
1
2
1a,b1a,b
D2O
G/ABC/GA
1
1
2
1a,b1a,b
GA
4
2
3
D2O
DMSO
DMSO
ABC
Glycerol
2
1a,b1a,b
14
13
12
11
10
9
8
7
6
5
4
3
2
1
0
f1 (ppm)

b.1)

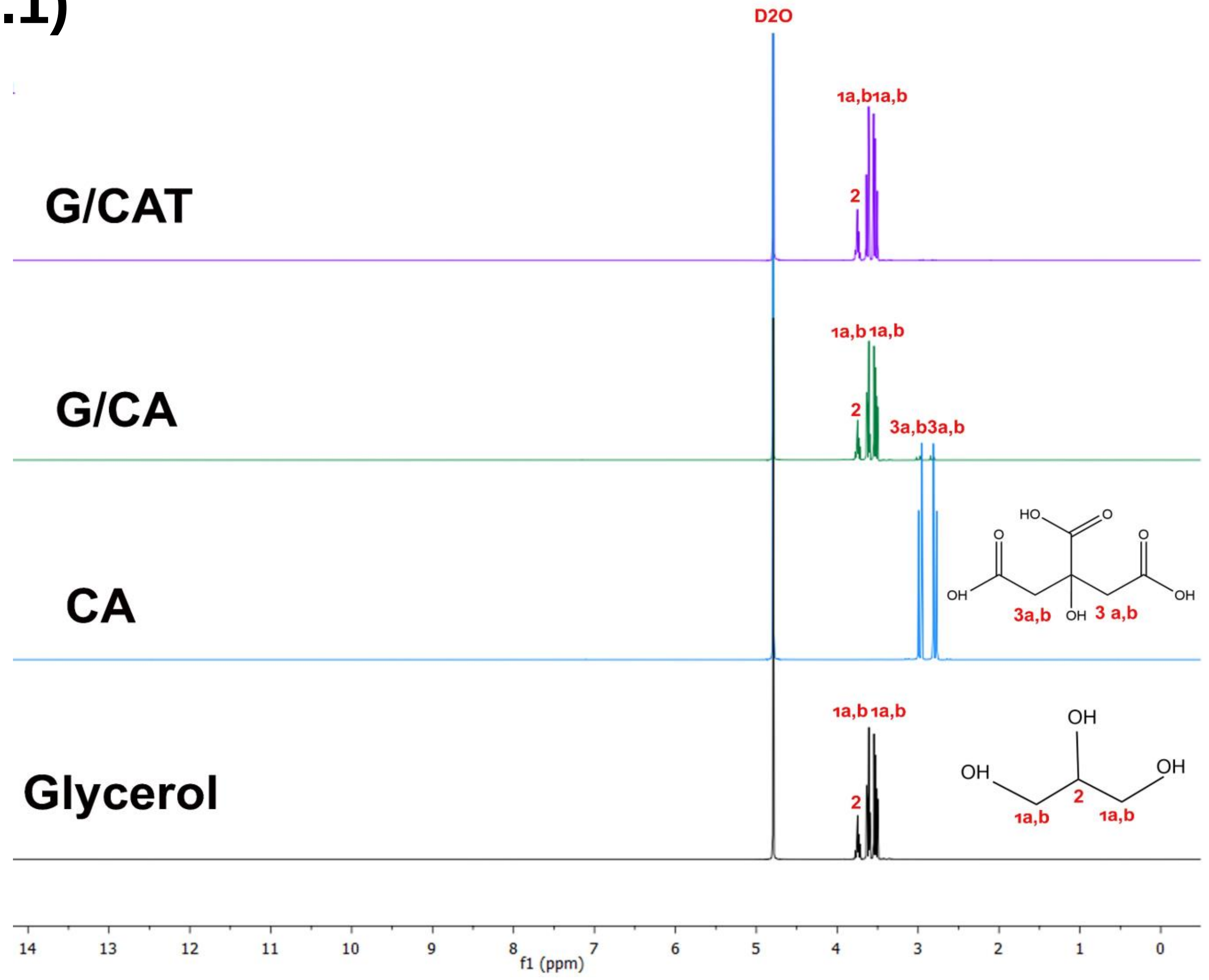
D2O
G/CAT
1a,b1a,b
2
G/CA
1a,b 1a,b
2
3a,b3a,b
CA
HO
O
OH
OH
OH
3a,b
3 a,b
Glycerol
1a,b 1a,b
2
OH
OH
OH
2
1a,b
1a,b
14 13 12 11 10 9 8 7 6 5 4 3 2 1 0
f1 (ppm)

b.2)

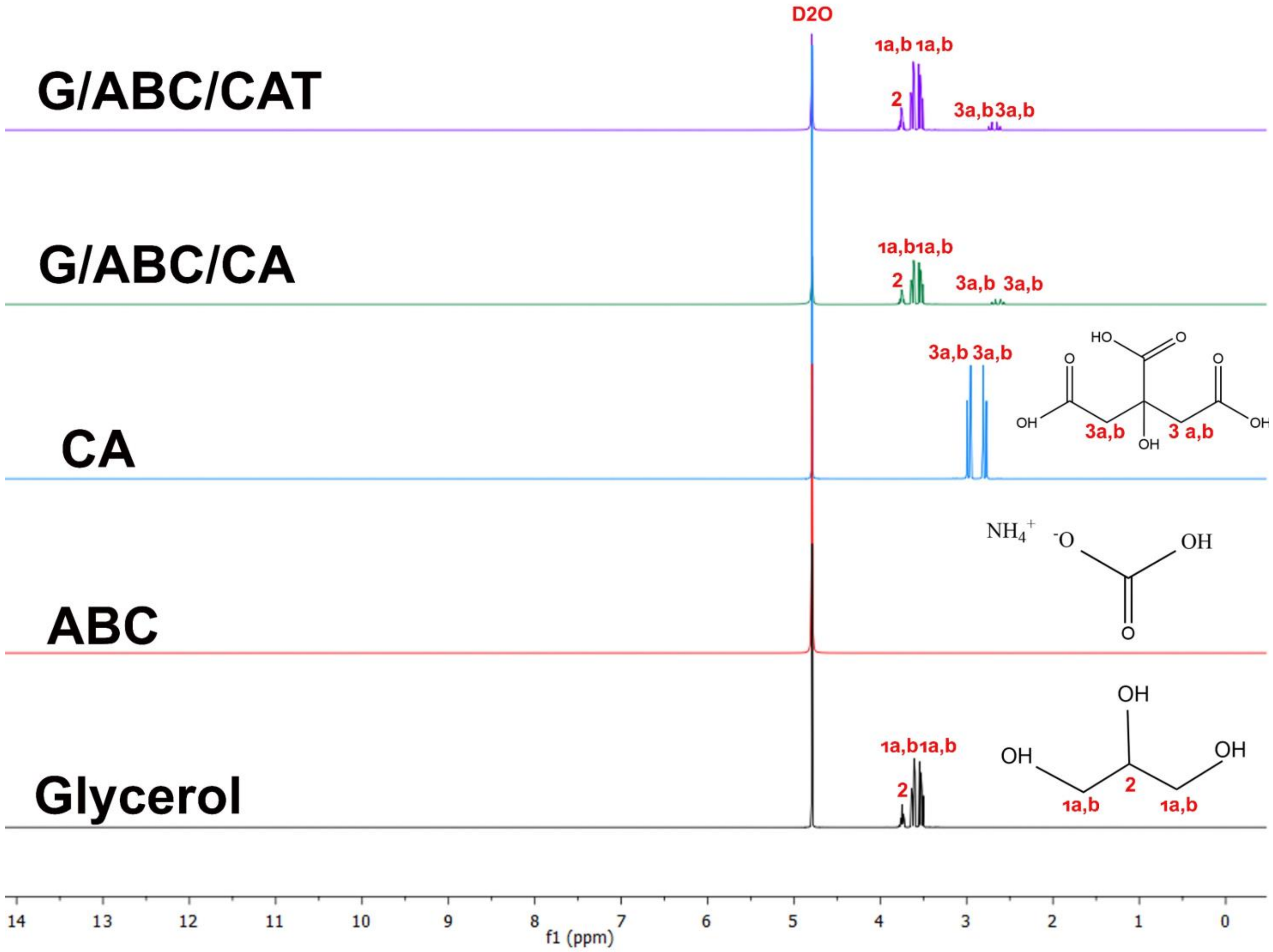
D2O
G/ABC/CAT
1a,b 1a,b
2
3a,b3a,b
G/ABC/CA
1a,b1a,b
2
3a,b 3a,b
CA
3a,b 3a,b
HO
O
OH
3a,b
OH
3 a,b
ABC
NH_4^+
^-O
OH
O
Glycerol
1a,b1a,b
2
OH
OH
OH
1a,b
2
1a,b
14
13
12
11
10
9
8
7
6
5
4
3
2
1
0
f1 (ppm)

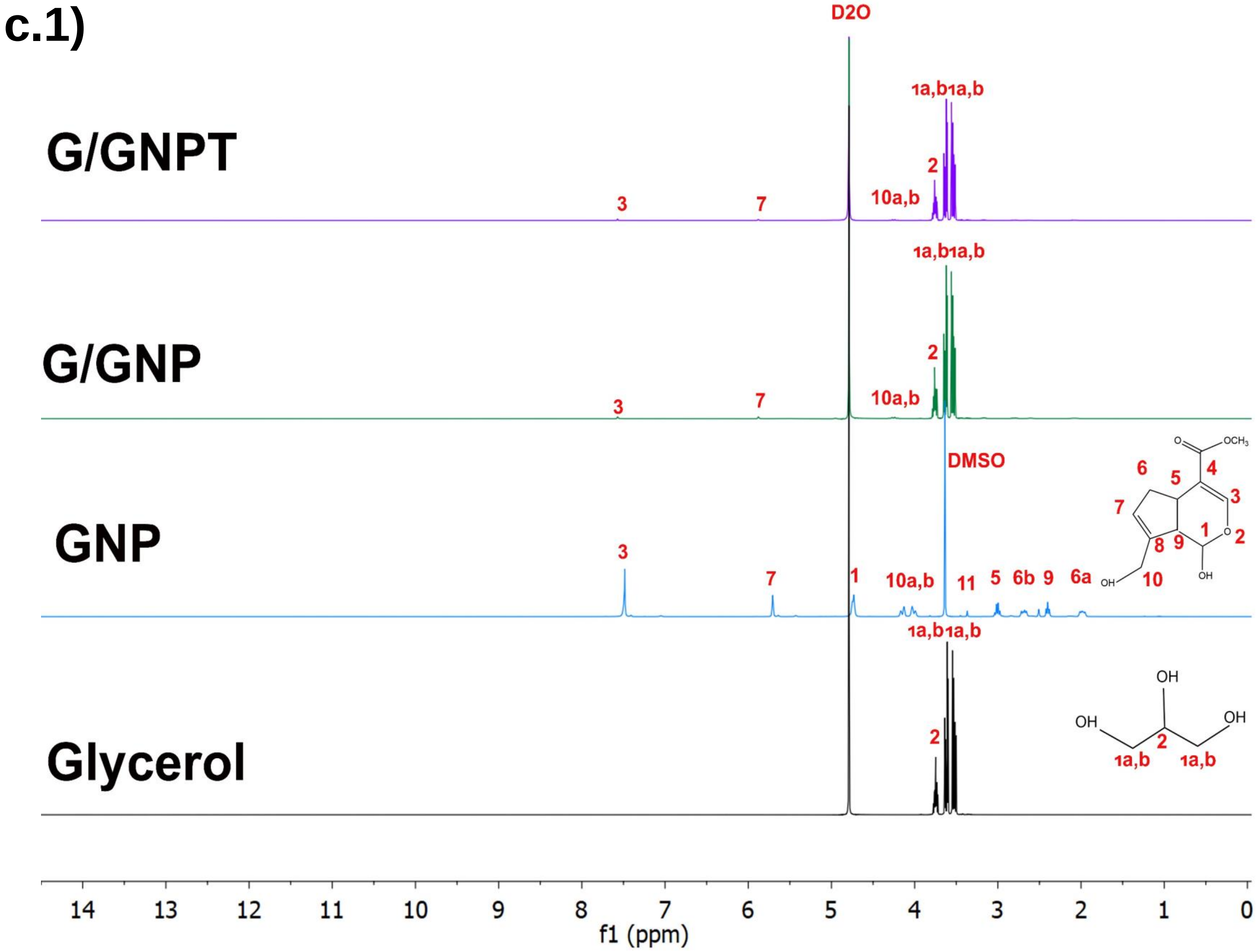
c.1)
D2O
G/GNPT
1a,b1a,b
2
10a,b
3
7
G/GNP
1a,b1a,b
2
10a,b
3
7
DMSO
GNP
3
7
1
10a,b
11
5
6b
9
6a
O
OCH3
6
5
4
3
7
1
2
8
9
OH
10
OH
Glycerol
1a,b1a,b
2
OH
OH
OH
2
1a,b
1a,b
14
13
12
11
10
9
8
7
6
5
4
3
2
1
0
f1 (ppm)

c.2)

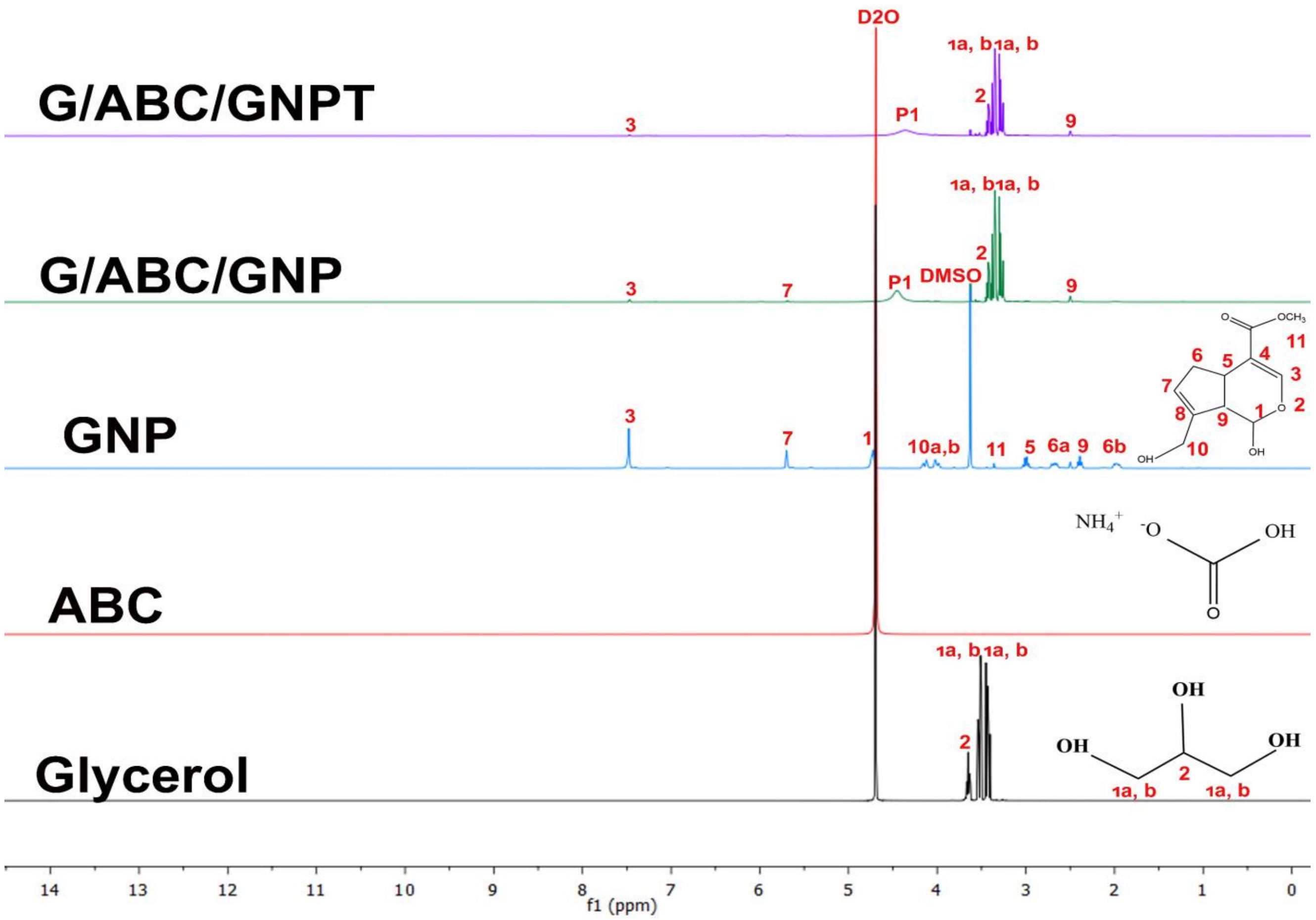

Supplementary Fig. S12. ^{1}H NMR spectra of the different mixtures and components. **a.1-a.3**, gallic acid, **b.1**,**b.2**, citric acid, and **c.1**,**c.2**, genipin systems.
