# Supplementary material for: Effects of multi-functional additives during foam extrusion of wheat gluten materials
Source: Commun Chem. 2024 Apr 3;7:75. doi: 10.1038/s42004-024-01150-1 (PMC10991538; doi:10.1038/s42004-024-01150-1)
Supplement: Supplementary file 3 — Description of Additional Supplementary Files [file 42004_2024_1150_MOESM3_ESM.pdf]

Description of Additional Supplementary Files

File name: Supplementary Video 1

Description: Determination of gas expansion with ammonium bicarbonate as a foaming agent.

File name: Supplementary Video 2

Description: Manufacturing of Wheat gluten foams.
